# The effect of age on cerebral blood flow responses during repeated and sustained stand to sit transitions

**DOI:** 10.14814/phy2.14421

**Published:** 2020-05-06

**Authors:** Timo Klein, Tom G. Bailey, Petra Wollseiffen, Stefan Schneider, Christopher D. Askew

**Affiliations:** ^1^ VasoActive Research Group School of Health and Sport Sciences University of the Sunshine Coast Maroochydore DC QLD Australia; ^2^ Institute of Movement and Neuroscience German Sport University Cologne Cologne Germany; ^3^ Centre for Research on Exercise Physical Activity and Health School of Human Movement and Nutrition Sciences The University of Queensland Brisbane QLD Australia; ^4^ Sunshine Coast Health Institute Sunshine Coast Hospital and Health Service Birtinya QLD Australia

**Keywords:** aging, cerebral perfusion, cerebral pressure–flow relationship, MCAv, middle cerebral artery, TCD, vascular function

## Abstract

**Introduction:**

Aging is associated with impaired cerebrovascular blood flow and function, attributed to reduced vasodilatory capacity of the cerebrovascular network. Older adults may also have an impaired relationship between changes in blood pressure and cerebral blood flow; however, previous reports conflict. This study aimed to compare the blood pressure and cerebral blood flow responses to both repeated and sustained stand‐to‐sit transitions in young and older adults, and to assess the relationship with cerebrovascular reactivity.

**Methods:**

In 20 young (age: 24 ± 4 years) and 20 older (age: 71 ± 7 years) adults we compared middle cerebral artery flow velocity (MCAv), end‐tidal partial pressure of carbon dioxide (P_ET_CO_2_), and blood pressure (mean arterial blood pressure [MAP]) during repeated stand‐to‐sit (10 s standing and 10 s sitting) and sustained stand‐to‐sit (3 min standing followed by 2 min sitting) transitions. Cerebrovascular reactivity to changes in carbon dioxide levels was assessed using a repeated breath‐hold test.

**Results:**

The % change in MCAv per % change in MAP (%∆MCAv/%∆MAP) was higher in the older adults than in the young adults during repeated stand‐to‐sit transitions. During the sustained protocol the %∆MCAv/%∆MAP response was similar in both age groups. A high %∆MCAv/%∆MAP response during the repeated stand‐to‐sit protocol was associated with low cerebrovascular reactivity to CO_2_ (*r* = −.39; *p* < .01), which was significantly lower in the older adults.

**Conclusion:**

These findings suggest that the higher %∆MCAv/%∆MAP during repeated stand–sit transitions was associated with impaired cerebrovascular reactivity. Impairments in endothelial function and vascular stiffness with age may contribute to the altered transient cerebral pressure–flow responses in older adults.

## Graphical abstract

Aging and age‐related cerebrovascular diseases are associated with impaired cerebrovascular function. The novel finding of this study is that older adults showed higher pressure–flow responses during repeated stand–sit transitions compared to younger adults, and that a high pressure–flow response was associated with low cerebrovascular reactivity to CO_2_. These results suggest that impaired vascular function and increased arterial stiffness may contribute to the pressure–flow responses observed in the older adults.

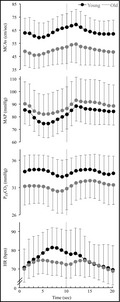

## 1 INTRODUCTION

Aging and age‐related cerebrovascular diseases are associated with a decline in resting cerebral blood flow, commonly characterized by a lower middle cerebral artery velocity (MCAv), and reduced cerebrovascular function (Ainslie et al., 2008; Bailey et al., 2013; Beek, Olde Rikkert, Pasman, Hopman, & Claassen, 2010). Furthermore, the transient elevation in MCAv during exercise is attenuated in older adults (Fisher et al., 2013; Fluck et al., 2014; Klein, Bailey, Abeln, Schneider, & Askew, 2019; Marsden et al., 2012). Cerebral blood flow control is multifactorial, and it is therefore difficult to isolate the relative regulatory contributions of neural, metabolic, and cardiovascular factors. Changes in mean arterial blood pressure (MAP) are believed to be a key driver for the dynamic MCAv response to exercise. However, in older adults the lower MCAv response to exercise is often reported in the presence of larger increases in MAP compared with young adults (Fisher et al., 2013; Fluck et al., 2014; Klein et al., 2019; Marsden et al., 2012). This raises the possibility that an impaired responsiveness to transient or repetitive increases in blood pressure may contribute to the lower cerebral blood flow with aging.

Sitting and squatting maneuvers have become popular for the assessment of the MCAv‐MAP (pressure–flow) relationship, and are favored because they are well tolerated, replicate activities of daily living and can be performed without significant changes in P_ET_CO_2_ (Claassen, Meel‐van den Abeelen, & Simpson, 2016). There are conflicting reports about the effect of age on the isolated MCAv‐MAP relationship, with some studies reporting no difference between age groups (Xing et al., 2017), and others reporting an altered flow response in older adults (Oudegeest‐Sander et al., 2014). Recently, it was reported that the relative response of MCAv to transient *increases* in blood pressure (%∆MCAv/%∆MAP) (squatting) was attenuated compared with the response to *decreases* in blood pressure (standing) during a repeated squat‐stand protocol in a cohort of men aged 20–74 years (Brassard et al., 2017). Moreover, increasing age was positively associated with the %∆MCAv/%∆MAP response during the hypertensive (squatting) phase, but not during the standing phase where there is a decrease in blood pressure. This may have implications for the influence of age on the MCAv response to dynamic *increases* in blood pressure, although to date there have been no direct comparisons between younger and older adults reported.

Any alterations in the pressure–flow relationship with age are likely to be attributed to alterations in vascular function. Aging is associated with impaired cerebrovascular reactivity, where older adults show a reduced MCAv response to a hypercapnic challenge (Bailey et al., 2013; Barnes, Taylor, Kluck, Johnson, & Joyner, 2013; Coverdale, Badrov, & Shoemaker, 2017). This impairment is largely attributed to a limitation in endothelial function and the vasodilatory capacity of the cerebrovascular network in older adults (Miller, Howery, Harvey, Eldridge, & Barnes, 2018; Xie et al., 2006). Such an impairment in cerebrovascular function might influence the pressure–flow relationship, although this has not been investigated. To address this, the current study aimed to compare the %∆MCAv/%∆MAP response to repeated stand‐to‐sit transitions, and to assess its relationship with cerebrovascular reactivity, between younger and older adults.

## 2 METHODS

### 2.1 Participants

Twenty young (age: 24 ± 4 years) and 20 older (age: 71 ± 7 years) adults participated in the study (Table 1). Participants were excluded if they had known hypertension, known diabetes mellitus, or a diagnosed cardiovascular or cerebrovascular condition. None of the participants were using prescribed or over‐the‐counter medications or were current smokers. All experimental procedures conformed to the *Declaration of Helskini* and were approved by the local ethics committee of the University of the Sunshine Coast (protocol number S16877). A detailed verbal and written explanation of the study was provided, and written informed consent was obtained from each participant before participation.

**Table 1:** Characteristics of young and older adults

	Young (*n* = 20)	Older (*n* = 20)	*p‐*value
Male:Female (*n*)	11:9	9:11	
Age (years)	24 ± 4	71 ± 7	<.001
Weight (kg)	68.4 ± 12.8	72.1 ± 12.8	.36
Height (m)	1.74 ± 0.08	1.71 ± 0.07	.16
BMI (kg m^−2^)	22 ± 3	25 ± 4	.03
Systolic blood pressure (mmHg)	127 ± 18	124 ± 26	.70
Diastolic blood pressure (mmHg)	54 ± 15	50 ± 12	.37

Note

Data are displayed as mean ± *SD*. BMI, body mass index.

### 2.2 Study overview

After screening and familiarization, participants attended the laboratory following an overnight fast and having refrained from alcohol and physical activity for 24 hr and caffeine for 12 hr. Participants were fitted with instruments to measure MCAv, MAP, P_ET_CO_2_, heart rate (HR), and cardiac output (CO), which were recorded throughout the study session. Resting measurements and cerebrovascular function (CO_2_ reactivity) were initially assessed. Participants then performed the sustained and repeated stand‐to‐sit protocols, respectively.

#### 2.2.1 Resting measures and breath‐hold test

Resting measurements were collected during a 5‐min period of supine rest before the cerebrovascular function test. Cerebrovascular function was assessed as cerebral blood flow (MCAv) reactivity to changes in carbon dioxide with a repeated breath‐hold test while in the supine position (Tancredi & Hoge, 2013). After paced breathing, participants held their breath for 20 s. A metronome was used as a guide for paced breathing and set at 16 breaths per minute for 30 s before the next breath‐hold started. Participants were instructed to give a small forced exhalation at the end of each breath‐hold. Breath‐holds were repeated eight times (Murphy, Harris, & Wise, 2011; Tancredi & Hoge, 2013).

#### 2.2.2 Stand‐to‐sit protocols

After resting measures, participants completed a 3‐min period of sustained standing followed by a 2‐min period of sitting. This was followed by the repeated stand‐to‐sit transition protocol, which consisted of 13 stand‐to‐sit transitions in a 5‐min period at a frequency of 0.05 Hz (10 s in the standing position and 10 s in the seated position). This same protocol has previously been used in older adults (den Abeelen, Lagro, Beek, & Claassen, 2014; Beek, Claassen, Rikkert, & Jansen, 2008; Beek et al., 2010; Oudegeest‐Sander et al., 2014). The angle of the left knee was continuously measured with a bipolar sensor (Goniometer, MLTS700, ADInstrument) to enable the alignment of data with each period of standing and sitting. The average stand‐to‐sit transition time was 1.83 ± 0.23 s, which did not differ between the young and older groups (*p *= .40).

### 2.3 Measurements

MCAv was assessed using transcranial Doppler ultrasonography (TCD, Multigon, Neurovision) by placing a 2 MHz probe over the temporal window. The left and right MCAv signals were identified and tested according to standardized criteria guided by signal depth, velocity, and wave characteristics (Aaslid, Markwalder, & Nornes, 1982; Willie et al., 2011). The side with the best signal quality, including the highest mean MCAv at rest, was used for testing. The ultrasound probe was fixed at a constant angle and secured with a headband (Multigon, Neurovision). The signal depth, sample volume, and power remained constant throughout the test session after establishing an optimal MCAv signal.

Blood pressure was measured continuously at the left middle finger using photophlethysmography (Finometer MIDI, Finapres Medical Systems). Participants placed their left hand in a sling across their chest to keep the hand at heart‐level. Finger blood pressure was exported to generate beat‐by‐beat systolic (SBP), diastolic (DBP) and mean arterial pressure (MAP), and HR and CO (ADInstruments, PowerLab 8/35).

Participants wore a leak‐free respiratory mask (Hans‐Rudolph, Kansas City, MO, USA) from which the breath‐by‐breath partial pressure of end‐tidal carbon dioxide (P_ET_CO_2_) was determined continuously (ADInstrument, Gas Analyser).

### 2.4 Data analysis

MCAv, MAP, P_ET_CO_2_, HR, CO, and goniometer data were simultaneously sampled at 1 kHz via an analog‐to‐digital converter and stored for offline analysis (LabChart Pro v8 and PowerLab, ADInstruments). Time‐aligned signals were resampled (smoothed) using second‐by‐second data (1 Hz) for analysis. Cerebrovascular resistance was calculated as MAP relative to MCAv.

#### 2.4.1 Sustained and repeated stand‐to‐sit analysis

Measures were averaged over the last 60 s of the supine rest period, and over the last 60 s of the sustained standing period and the sustained seated period. These average responses were used to calculate the *sustained* stand‐to‐sit responses (i.e., delta: sit–stand).

During each *repeated* stand‐to‐sit transition, the smoothed second by second data were used to identify maximum values during the sit phase, and minimum values during the stand phase, for MCAv, MAP, and P_ET_CO_2_. Whereas for HR and CO responses, maximum values were identified during the stand phase and minimum values during each sit phase. For each transition, the response of each variable was calculated using the delta (sit–stand). Time to maximum and minimum responses during the repeated stand‐to‐sit transitions were calculated for MAP and MCAv. The transition delta responses were also expressed as a relative percentage of the stand phase values (%∆), and the ratio of %∆MCAv/%∆MAP was calculated for each transition as previously reported (Brassard et al., 2017). For each variable, responses during each of the 13 stand‐to‐sit transitions were compared, and an average across all transitions was also calculated for further comparison between age groups.

#### 2.4.2 Cerebrovascular reactivity

The breath‐hold test is a validated test to assess cerebrovascular function (Tancredi & Hoge, 2013). For each breath‐hold maneuver, the increase in P_ET_CO_2_ (∆P_ET_CO_2_) was calculated by subtracting the average of the last two breaths before the breath‐hold from the peak P_ET_CO_2_ response immediately after the breath‐hold. Breath‐holds were analyzed separately and a total of six breath‐holds per person were averaged to compare the responses between groups. The first two breath‐hold maneuvers were used to familiarize participants with the task (Tancredi & Hoge, 2013). Cerebrovascular reactivity was calculated as the increase in MCAv relative to the corresponding increase in P_ET_CO_2_ as absolute (∆MCAv/∆P_ET_CO_2_) and relative (%∆MCAv/∆P_ET_CO_2_) responses.

### 2.5 Statistical analysis

The effect of age on responses during sustained standing and sitting and on cerebrovascular CO_2_ reactivity was determined by comparing the results from the young and older groups using an independent *t*‐test. Two‐factor (age * time) ANOVA for repeated measures was used to compare the MCAv, MAP, P_ET_CO_2_, HR, CO, and %∆MCAv/%∆MAP responses of the two groups (young and older) over the repeated stand‐to‐sit transitions (time: from transition 1 to transition 13). Significant main effects and interactions were followed up with post hoc Bonferroni comparisons. Averaged MCAv, MAP, P_ET_CO_2_, HR, CO, time to peak MAP and MCAv, and %∆MCAv/%∆MAP responses during the repeated stand‐to‐sit transitions were further compared with an independent *t*‐test. Pearson correlation coefficients were used to examine the relationships between the average %∆MCAv/%∆MAP response during the repeated stand‐to‐sit protocol and age, %∆P_ET_CO_2_, %∆HR, %∆CO, and cerebrovascular CO_2_ reactivity; as well as the relationship between the %∆MCAv/%∆MAP response during sustained stand‐to‐sit and cerebrovascular CO_2_ reactivity. Statistical significance was set at *p *<.05*.* Statistical analyses were performed with Statisitca 7.1. (StatSoft).

## 3 RESULTS

Resting blood pressure was similar between age groups. BMI was significantly greater in the older group than in the young group (Table 1). Baseline MCAv during the sustained stand and sit positions was significantly higher in the young group than in the older group, but the MAP during both positions was similar in both groups (Table 2).

**Table 2:** Average responses during periods of sustained stand and sustained sit in young and older adults

	Stand		Sit		∆ (Sit − Stand)	
	Young	Older	Young	Older	Young	Older
MCAv (cm s^−1^)	63.0 ± 10.4	49.2 ± 9.6*	64.2 ± 10.3	50.0 ± 9.9*	1.1 ± 3.2	0.8 ± 2.4
MAP (mmHg)	80.7 ± 11.0	87.0 ± 14.7	79.5 ± 10.2	80.8 ± 15.7	−1.2 ± 4.1	−2.3 ± 7.6
P_ET_CO_2_ (mmHg)	31.1 ± 3.4	28.4 ± 6.8	31.6 ± 4.3	30.2 ± 3.1	0.5 ± 1.6	0.0 ± 1.8
Heart rate (beats min^−1^)	77.2 ± 11.7	69.9 ± 6.7*	69.6 ± 9.4	65.7 ± 6.1	−7.5 ± 5.1	−4.3 ± 3.5
Cardiac output (L min^−1^)	5.4 ± 1.1	3.5 ± 0.9*	5.6 ± 1.0	4.1 ± 2.5*	0.1 ± 0.6	0.3 ± 1.4
mmHg^−1^ cm s^−1^)	1.27 ± 0.25	1.74 ± 0.60*	1.31 ± 0.28	1.81 ± 0.57*	‐0.05 ± 0.09	−0.06 ± 0.17
%∆MCAv/ %∆MAP (%%)	–	–	–	–	0.38 ± 2.9	−0.1 ± 1.7

Note

Data are displayed as mean ± *SD*. Data represent the average of the last 60 s during 3 min standing and during 2 min sitting, respectively.

Abbreviations: CVR, cerebrovascular resistance; MAP, mean arterial blood pressure; MCAv, middle cerebral artery flow velocity; P_ET_CO_2_, partial pressure of end‐tidal carbon dioxide.

* Significant difference between young and older groups (*p* < .05).

The change in P_ET_CO_2_ from baseline to peak during the cerebrovascular reactivity test was similar in the young and older groups (Table 3), but the absolute and relative cerebrovascular CO_2_ reactivity was lower in the older group than in the young group.

**Table 3:** Cerebrovascular reactivity measured during breath‐hold test in young and older adults

	Baseline		Peak response during breath‐hold		∆ (Peak − Baseline)	
	Young	Older	Young	Older	Young	Older
MCAv (cm s^−1^)	62.3 ± 11.6	43.1 ± 10.5*	78.9 ± 13.2*	53.8 ± 12.5*	16.6 ± 7.2	10.7 ± 3.4*
MAP (mmHg)	71.8 ± 10.0	71.1 ± 17.1	78.8 ± 11.7	75.2 ± 18.2	7.1 ± 5.1	4.2 ± 3.6
P_ET_CO_2_ (mmHg)	33.5 ± 3.3	28.3 ± 3.4*	39.6 ± 3.9	35.1 ± 4.1*	6.1 ± 1.5	6.8 ± 1.6
CVR (mmHg^−1^ cm s^−2^)	1.18 ± 0.22	1.81 ± 0.83*	1.02 ± 0.20	1.52 ± 0.69*	−0.16 ± 0.07	−0.28 ± 0.15*
CO_2_ reactivity (cm s^−1^ mmHg^−1^)	—	—	—	—	3.0 ± 1.3	1.7 ± 0.7*
CO_2_ reactivity (%cm s^−1^ mmHg^−1^)	—	—	—	—	5.1 ± 2.1	3.9 ± 1.3*

Note

Data are displayed as mean ± *SD*. Data represent the average responses of six consecutive breath‐holds.

Abbreviations: CO_2_ reactivity, cerebrovascular reactivity; CVR, cerebrovascular resistance; MAP, mean arterial blood pressure; MCAv, middle cerebral artery flow velocity; P_ET_CO_2_, partial pressure of end‐tidal carbon dioxide.

* Significant difference between young and older groups (*p *< 0.05).

### 3.1 Repeated stand‐to‐sit responses

**Figure 1:**
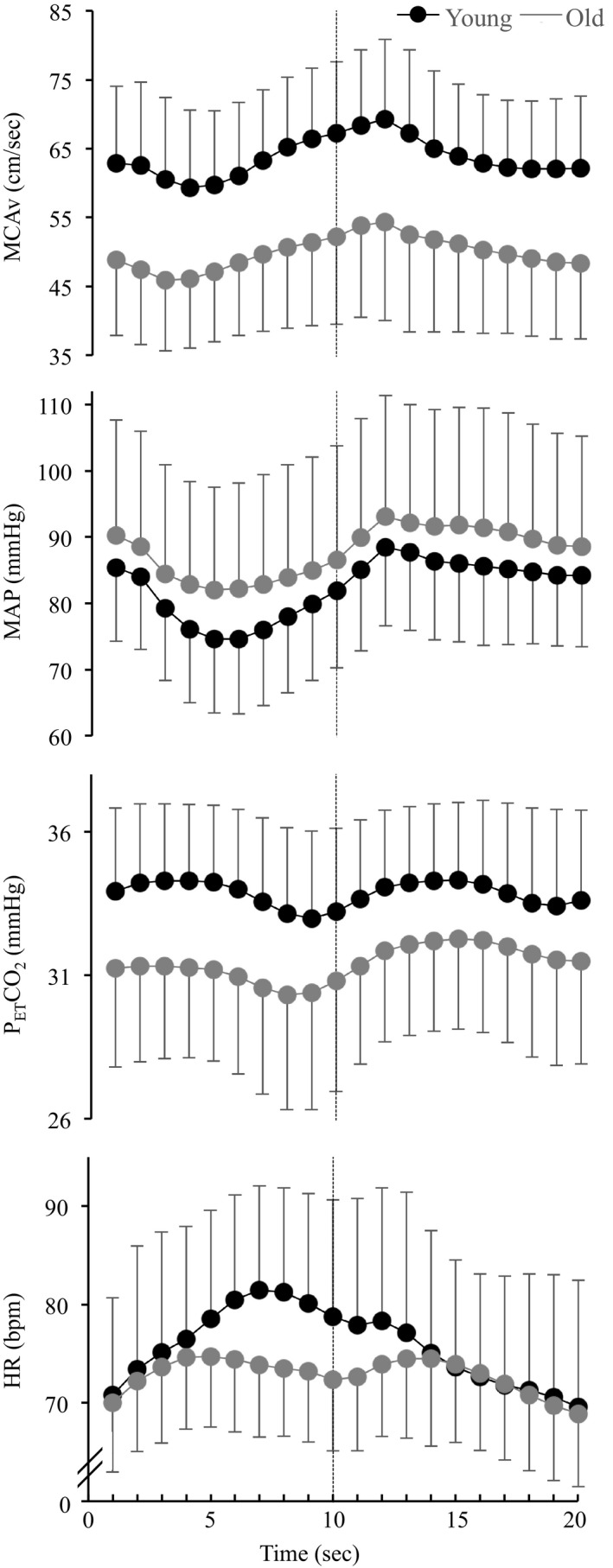
Cardiovascular and cerebrovascular responses during repeated stand‐to‐sit transitions in young and older adults. Averaged second by second data (1 Hz) are shown for the repeated stand‐to‐sit transitions for the Young (black dots) and Old groups (grey dots). The first phase represents the standing position (1–10 s) and the second phase represents the sitting position (11–20 s). MCAv, middle cerebral artery flow velocity; MAP, mean arterial blood pressure; P_ET_CO_2_, partial pressure of end‐tidal carbon dioxide; HR, heart rate. Data are displayed as mean ± *SD*

**Figure 2:**
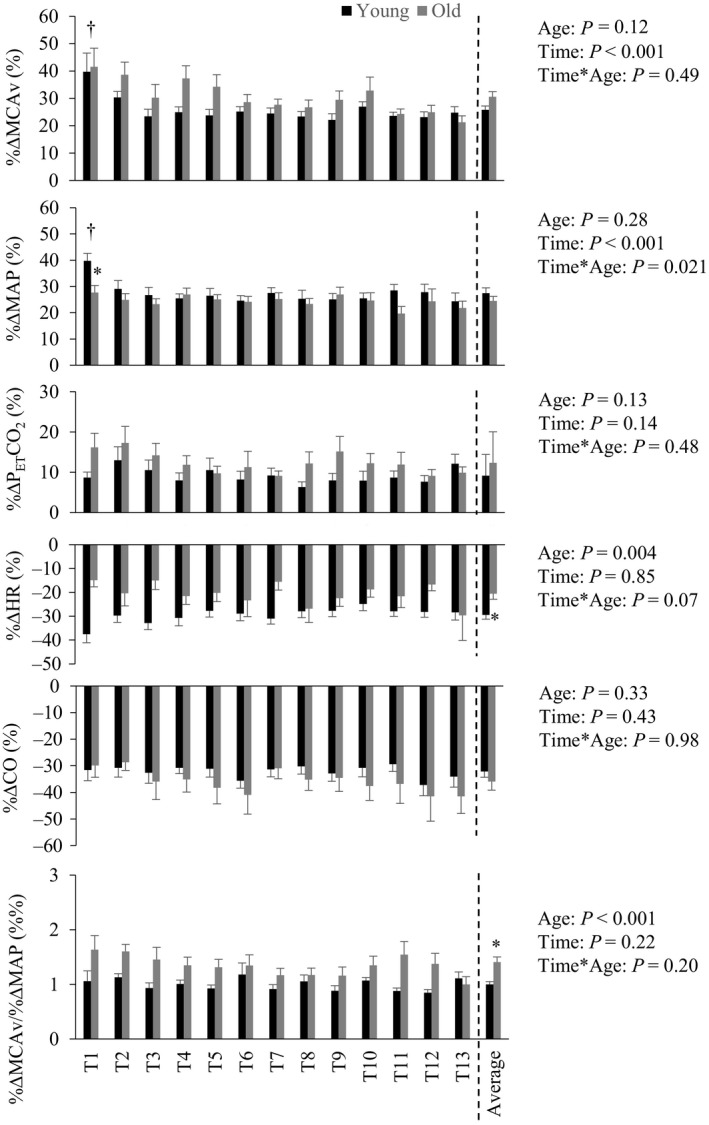
Cardiovascular and cerebrovascular responses during repeated stand‐to‐sit transitions in young and older adults. Participants performed 13 repeated transitions from a standing position (10 s) to a seated position (10 s). Relative changes from stand to sit positions for each of the transitions (T1–T13) are shown. Average response of all transitions are also shown (right columns). MCAv, middle cerebral artery flow velocity; MAP, mean arterial blood pressure; P_ET_CO_2_, partial pressure of end‐tidal carbon dioxide; HR, heart rate; CO, cardiac output. Group × transition effects were assessed using repeated‐measures ANOVA. Average responses were compared using an independent *t*‐test. *p < .01 difference between young and old, ^†^ *p *< .05 different from other transitions for both young and old. Data are displayed as mean ± *SD*

Mean second by second responses during the repeated stand‐to‐sit transitions are shown in Figure 1, and the average delta (sit–stand) response for each variable is shown in Table 4. Stand‐to‐sit transitions are also shown as relative changes from the standing position in Figure 2. Average increases in %∆MAP and %∆MCAv across the repeated stand‐to‐sit transitions did not differ between the groups. Time to peak from standing to sitting for MAP (young: 8.5 ± 1.4 s vs. old: 8.5 ± 1.2 s; *p* = .94) and MCAv (young: 8.7 ± 1.4 s vs. old: 9.2 ± 1.3 s; *p* = .25) was not different between young and old. As shown in Figure 2, the first transition (T1) caused the greatest increase in %∆MCAv and %∆MAP. The effect then stabilized for the subsequent transitions and this response was similar in both groups. The repeated stand‐to‐sit transitions resulted in a higher average %∆MCAv/%∆MAP in the older group than in the young group (*p *<.001, Figure 2 and Table 4). While the absolute increase in CVR during repeated stand‐to‐sit transitions was greater in the older group (Table 4), the relative increase in CVR was not different between age groups (young: 24.3 ± 7.8% vs old: 28.2 ± 10.7%; *p* = .20). The slope of the linear relationship between %∆CVR and %∆MAP for the averaged repeated stand‐to‐sit responses was 0.74 in the young (*r* = .68; *p* < .001) and −0.10 (*r* = −.15; *p* = .54) in the older adults.

**Table 4:** Average responses during repeated stand‐to‐sit transitions in young and older adults

	Stand		Sit		Transition (∆Sit − Stand)	
	Young	Older	Young	Older	Young	Older
MCAv (cm s^−1^)	57.3 ± 10.5	43.9 ± 8.3*	71.1 ± 11.3	56.8 ± 12.1*	14.1 ± 3.5	12.9 ± 5.2
MAP (mmHg)	73.3 ± 10.7	81.2 ± 16.5	91.7 ± 10.8	99.7 ± 17.8	19.0 ± 4.4	18.8 ± 5.7
P_ET_CO_2_ (mmHg)	32.2 ± 2.5	28.9 ± 6.9	34.8 ± 2.2	32.3 ± 7.1	2.7 ± 1.4	3.3 ± 1.8
Heart rate (beats min^−1^)	85.1 ± 9.7	78.2 ± 5.8*	66.2 ± 9.9	66.2 ± 5.9	–18.7 ± 3.6	–12.4 ± 5.6*
Cardiac output (L min^−1^)	7.8 ± 1.5	5.1 ± 1.8*	6.0 ± 1.2	3.8 ± 1.1*	–1.9 ± 0.6	–1.6 ± 1.2
CVR (mmHg^−1^ cm s^−2^)	1.18 ± 0.2	1.67 ± 0.60*	1.47 ± 0.28	2.16 ± 0.79*	0.28 ± 0.10	0.47 ± 0.27*
%∆MCAv/ %∆MAP (%%)	—	—	—	—	1.0 ± 0.19	1.6 ± 1.3*

Note

Data are displayed as mean ± *SD*. Data represent the average responses for the stand‐to‐sit transitions.

Abbreviations: CVR, cerebrovascular resistance; MAP, mean arterial blood pressure; MCAv, middle cerebral artery flow velocity; P_ET_CO_2_, partial pressure of end‐tidal carbon dioxide.

* Significant difference between young and older groups (*p* < .05).

### 3.2 Correlations

Age was positively correlated with the mean %∆MCAv/%∆MAP response during the repeated stand‐to‐sit transitions (*r* = .50; *p* < .001), with the higher responses in the older participants. In the young group, the mean %∆MCAv/%∆MAP response to the repeated stand‐to‐sit transitions was not significantly correlated with P_ET_CO_2_ (*r* = .39; *p* = .08), HR (*r* = –.07; *p *= .75) or CO (*r* = .04; *p *= .84). Similarly, in the older group, %∆MCAv/%∆MAP was not correlated with P_ET_CO_2_ (*r* = –.21, *p *= .35), HR (*r* = .14; *p *= .55) or CO (*r* = .16; *p *= .49). Mean %∆MCAv/%∆MAP during repeated stand‐to‐sit transitions was inversely correlated with cerebrovascular reactivity (∆MCAv/∆P_ET_CO_2_) when examining the full study cohort (*n* = 40, *r* = –.39; *p *<.01), but not when considering the young (*r* = –.19; *p *= .40) and older (*r* = –.13; *p *= .57) groups separately. Mean %MCAv/%MAP response to the sustained stand‐to‐sit transition was not correlated with cerebrovascular reactivity in the full study cohort (*n* = 40, *r* = −.002; *p* = .99), and not when considering the young (*r* = –.05; *p *= .84) and older (*r* = –.008; *p *= .97) groups separately.

## 4 DISCUSSION

This study aimed to compare the %∆MCAv/%∆MAP responses to repeated and sustained stand‐to‐sit transitions, and to assess the relationship with cerebrovascular reactivity, between young and older adults. The %∆MCAv/%∆MAP response during *sustained* stand‐to‐sit transition was similar in young and older adults, which agrees with others’ findings (Beek et al., 2008; Lipsitz, Mukai, Hamner, Gagnon, & Babikian, 2000; Oudegeest‐Sander et al., 2014; Sorond, Khavari, Serrador, & Lipsitz, 2005). The repeated rises in blood pressure during the rapid, intermittent stand‐to‐sit maneuvers resulted in a higher %∆MCAv/%∆MAP response in older adults than in young adults. This key finding suggests that older adults respond with a greater relative increase in cerebral blood flow for a given increase in blood pressure during repeated stand‐to‐sit transitions. Furthermore, the %∆MCAv/%∆MAP response during repeated stand‐to‐sit transitions was inversely correlated with cerebrovascular CO_2_ reactivity across the full study cohort. These findings indicate that impaired cerebral endothelial function in older adults might be associated with a disrupted dynamic cerebral pressure–flow relationship.

The primary aim of this study was to investigate the effect of age on the cerebral pressure–flow relationship during repeated stand‐to‐sit transitions. Our main interest in this was to better understand the smaller increase in MCAv, relative to the greater increase in blood pressure, that is consistently reported during exercise in older people (Fisher et al., 2013; Fluck et al., 2014; Klein et al., 2019; Ward et al., 2018). Repeated stand‐to‐sit transitions reflect a common daily movement and are a well‐accepted method to induce dynamic blood pressure changes, which we also demonstrated (Figure 1) (Claassen et al., 2016). However, the impact of age during transient stand‐to‐sit transitions differed to that reported during exercise, in that the MCAv of older adults increased more for a given change in MAP than that of young adults (Figure 2). Therefore, our observations during repeated stand‐to‐sit transitions are not able to explain the impaired MCAv response in older adults during dynamic aerobic exercise.

It is likely that the higher %∆MCAv/%∆MAP in the older group reflects an attenuated, and potentially slower, vasoconstriction effect in response to the blood pressure changes during repeated stand‐to‐sit transitions. Aging is generally associated with an increase in cerebrovascular resistance and reduced cerebral blood flow (Hart, Joyner, Wallin, & Charkoudian, 2012). Estimated cerebrovascular resistance in this study was higher in the older group, and it increased to a greater extent during the stand‐to‐sit transitions, than in the young (Table 4). This possibly reflects remodeling of the cerebral vasculature and is consistent with arterial stiffening in older adults (Donato, Machin, & Lesniewski, 2018; Fluck et al., 2014). It has been suggested that such remodeling of the cerebral vasculature in older adults may be a compensatory response, where the increase in vascular resistance protects downstream capillary beds and vulnerable brain tissues from hyper‐perfusion (Duffin et al., 2018).

In response to spontaneous blood pressure fluctuations or prolonged changes in blood pressure, it has generally been shown that *static* cerebral autoregulation is unaffected by age (Beek et al., 2008; Lipsitz et al., 2000; Sorond et al., 2005). Our finding that the relative MAP and MCAv responses to sustained stand‐to‐sit transitions did not differ between age groups also provides some support of an intact static autoregulatory mechanism in the older adults. However, there is a growing body of evidence that *dynamic* cerebral autoregulation is negatively influenced by age (Brassard et al., 2017; Oudegeest‐Sander et al., 2014; Smirl, Hoffman, Tzeng, Hansen, & Ainslie, 2016). Furthermore, age has been reported to be correlated with the dynamic pressure–flow relationship by Oudegeest‐Sander et al. (2014) (*r* = .28, *p* = .04), and independently by (Brassard et al. (2017) (*r* = .34; *p* = .01). In the young group, the slope of the relationship between %∆CVR and %∆MAP (slope = 0.74; *r* = .68; *p* < .001) during the repeated stand‐to‐sit maneuvers is in agreement with previous findings (Liu et al., 2013; Sorond et al., 2005), and reflects intact dynamic cerebral autoregulation compared with the older group (slope = −.10; *r* = −.15; *p* = .54). Studies of dynamic autoregulation typically assess the cerebral blood flow response to positive *and* negative fluctuations in blood pressure (Claassen et al., 2016). Given the recent report of a hysteresis effect, where the MCAv response to increases in blood pressure was attenuated compared with the response to decreases in blood pressure (Brassard et al., 2017), we specifically assessed the effects of repeated increases in blood pressure during the stand‐to‐sit transitions. Therefore, while the aim of this study was not to assess cerebral autoregulation per se, our findings provide some support that dynamic autoregulation is impaired in older adults, and this likely contributes to the higher %∆MCAv/%∆MAP response during the repeated stand‐to‐sit test.

Besides changes to vascular structure, with age there are also well‐established changes in vascular function, including the development of endothelial dysfunction that is attributed to inflammation and oxidative stress (Donato et al., 2018). Impairments in cerebral endothelial function with age may contribute to the higher %∆MCAv/%∆MAP response in older adults. Consistent with others (Bailey et al., 2013; Bakker et al., 2004; Barnes, Taylor, Kluck, et al., 2013), we observed a lower cerebrovascular CO_2_ reactivity in the older group compared with the young (Table 3). Importantly, we also found a significant association between CO_2_ reactivity and the %∆MCAv/%∆MAP response to the repeated stand‐to‐sit maneuvers. The endothelium plays an important role in regulating cerebral blood flow, where nitric oxide synthase inhibition leads to constriction of the cerebral arteries, both in vitro and in vivo, and decreases cerebral blood flow (Faraci, 1991; Prado, Watson, Kuluz, & Dietrich, 1992; You, Johnson, Marrelli, Mombouli, & Bryan, 1999). With age, the dampened flow‐velocity response to CO_2_ is likely due to impaired dilation of the cerebral arteries (Coverdale et al., 2017), and particularly the downstream microvasculature. It has previously been shown that cerebrovascular CO_2_ reactivity is impaired in patients with systemic endothelial dysfunction, and that this impairment is highly dependent on the availability of nitric oxide (Lavi, Gaitini, Milloul, & Jacob, 2006). As such, the significant association with CO_2_ reactivity in this study suggests that the %∆MCAv/%∆MAP response to repeated stand‐to‐sit maneuvers is possibly reliant on nitric oxide‐dependent endothelial function. Further investigation of the cerebral artery diameter changes in response to the stand‐to‐sit test, and other associated mechanisms, are needed to confirm this.

Given that the changes in MCAv during the repeated stand‐to‐sit test are quite rapid (<10 s), this raises the possibility that neural mechanisms may also explain the differences seen between the age groups. Age‐related elevations in sympathetic nerve activity augment cerebrovascular stiffness (Dinenno, Jones, Seals, & Tanaka, 2000), and potentially contribute to an impairment in the ability to regulate blood flow during the stand‐to‐sit transitions. Neurovascular coupling also contributes to the dynamic regulation of cerebral blood flow, and there is evidence of reduced hemodynamic responses to neural activation with age (Gauthier et al., 2013). Whether an age‐related impairment in neurovascular coupling explains the current findings is not clear. Interestingly, in animal models it has been shown that oxidative stress may impair the nitric oxide signaling from neurons to vessels with age (Lourenco, Ledo, Caetano, Barbosa, & Laranjinha, 2018), and this raises a potential link with the previously described endothelial dysfunction in older adults. Finally, impaired baroreceptor sensitivity in older adults might also explain the higher %∆MCAv/%∆MAP during repeated stand‐to‐sit transitions. Stiffening of barosensory arteries with age can blunt the sensitivity of baroreceptor function (Monahan et al., 2001; Okada et al., 2012), which in turn may contribute to the altered relationship of arterial blood pressure and cerebral blood flow, as observed during the transient rapid stand‐to‐sit protocol in the older group. Xing and colleagues (Xing et al., 2017) showed that baroreflex sensitivity was significantly attenuated in older adults compared to young adults. They proposed that baroreceptor sensitivity may contribute to better blood pressure control in young participants, and therefore reduced demand on dynamic cerebral autoregulation during sit–stand maneuvers.

While repeated stand‐to‐sit maneuvers caused large increases in blood pressure that are likely to be the primary driver of the MCAv response, we also observed changes in P_ET_CO_2_, HR, and CO (Figure 2) that may influence the response (Ainslie & Duffin, 2009; Smith & Ainslie, 2017). The 3 mmHg change in P_ET_CO_2_ probably accounts for ~ 9% of the increase in MCAv from standing, based on the assumption that MCAv increases by 3% for each mmHg rise in P_a_CO_2_ (Barnes, Taylor, & Nicholson, 2013; Willie et al., 2012). However, this would only explain about one‐third of the measured MCAv response during the stand‐to‐sit transitions. As the P_ET_CO_2_ response was similar in the young and older groups, and no relationship was found between %∆MCAv/%∆MAP and P_ET_CO_2_ for either group, it is unlikely that the differences in the MCAv response between age groups can be attributed to the changes in P_ET_CO_2_. HR and CO decrease during the transition from standing to sitting, and so are unlikely to explain the higher %∆MCA/%∆MAP in the older group. In summary, although we observed changes in P_ET_CO_2_, HR, and CO during repeated stand‐to‐sit transitions, the different response of %∆MCAv/%∆MAP in the young and older participants is not likely to be explained by these factors.

### 4.1 Limitations

There are some limitations in this study to consider. Currently, TCD is the only practical measure of intracranial cerebral blood flow velocity during stand‐to‐sit maneuvers. This approach assumes that the cross‐sectional diameter of the MCA remains constant; hence increases in blood flow velocity are assumed to be proportional to increases in cerebral blood flow. Although dilation of the MCA does occur during marked elevations (>15 mmHg) in P_ET_CO_2_ (Verbree et al., 2014), in our study we found relatively small maximum changes in P_ET_CO_2_ during the measurement of cerebrovascular function (6.5 ±1.5 mmHg) and stand‐to‐sit transitions (3.0 ±1.6 mmHg). Thus, changes in MCAv were unlikely to be influenced by changes in MCA diameter during this study. Although we tried to balance numbers of females and males in each age group, this study did not have the statistical power to compare responses between the sexes. Such a comparison would be further complicated by the known influence of the menstrual cycle and hormone replacement therapy on resting MCAv (Peltonen et al., 2016). We did not control for these factors and there is a need to further study sex differences in follow‐up investigations.

## 5 CONCLUSION

In conclusion, the %∆MCAv/%∆MAP responses during repeated stand‐to‐sit maneuvers were higher in older than in young adults, but the cerebrovascular reactivity to CO_2_ of the older adults was lower. The higher %∆MCAv/%∆MAP during repeated stand‐to‐sit transitions was associated with low cerebrovascular reactivity to CO_2_. We suggest that impaired vascular function and increased arterial stiffness likely contribute to the altered pressure–flow responses observed in the older adults.

## CONFLICT OF INTEREST

The authors declare no competing interests.
